# How Do Landscape Structure, Management and Habitat Quality Drive the Colonization of Habitat Patches by the Dryad Butterfly (Lepidoptera: Satyrinae) in Fragmented Grassland?

**DOI:** 10.1371/journal.pone.0138557

**Published:** 2015-09-16

**Authors:** Konrad Kalarus, Piotr Nowicki

**Affiliations:** Institute of Environmental Sciences, Jagiellonian University, Kraków, Poland; Deakin University, Australia, AUSTRALIA

## Abstract

Most studies dealing with species distribution patterns on fragmented landscapes focus on the characteristics of habitat patches that influence local occurrence and abundance, but they tend to neglect the question of what drives colonization of previously unoccupied patches. In a study of the dryad butterfly, we combined classical approaches derived from metapopulation theory and landscape ecology to investigate the factors driving colonization from a recent refugium. In three consecutive transect surveys, we recorded the presence and numbers of imagos in 27 patches of xerothermic grassland and 26 patches of wet meadow. Among the predictors affecting the occurrence and abundance of the dryad, we considered environmental variables reflecting (i) habitat patch quality (e.g., goldenrod cover, shrub density, vegetation height); (ii) factors associated with habitat spatial structure (patch size, patch isolation and fragmentation); and (iii) features of patch surroundings (100-m buffers around patches) that potentially pose barriers or provide corridors. Patch colonization by the dryad was strongly limited by the distance from the species refugium in the region; there was a slight positive effect of shrub density in this respect. Butterfly abundance increased in smaller and more fragmented habitat patches; it was negatively impacted by invasive goldenrod cover, and positively influenced by the density of watercourses in patch surroundings. Nectar plant availability was positively related to species abundance in xerothermic grassland, while in wet meadow the effect was the reverse. We conclude that dryad colonization of our study area is very recent, since the most important factor limiting colonization was distance from the refugium, while the habitat quality of target patches had less relevance. In order to preserve the species, conservation managers should focus on enhancing the quality of large patches and should also direct their efforts on smaller and more fragmented ones, including those with relatively low resource availability, because such habitat fragments have an important role to play for specialist species.

## Introduction

The persistence of many species on the landscape scale is related to land use and landscape configuration, especially as they influence the distribution of resources affecting habitat quality [1–3]. Fragmentation, leading to the loss of suitable habitat and to the deterioration of remaining habitat fragments, reduces the likelihood of species survival [4], [5]. In Europe, fragmentation is mainly the result of degradation of natural sites, invasions of alien species, and changes in agricultural practices leading either to landscape homogenization by large-scale agriculture or to natural succession due to abandonment of previously managed land, especially grassland [1], [6], [7]. Assessing habitat suitability and making good habitats more accessible for target species of conservation interest are major tasks for conservation biology [8–12].

The methodology of research on the spatial patterns of species distributions owes much to recent achievements of metapopulation and landscape ecology e.g. [13–15]. According to classic metapopulation theory, the landscape contains discrete habitat patches separated by a uniform inhospitable environment, called the matrix [16–18]; the landscape is assumed to form a black and white mosaic, and other spatial effects are neglected [16]. On the other hand, landscape ecology and more sophisticated metapopulation concepts consider the spatial variation of habitat quality and landscape heterogeneity, including compositional and spatial structure heterogeneity [15], [19–22]. From the perspective of particular species, the landscape may consist of a patchwork of various habitat types which offer different resources for foraging and reproduction [12], [23], [24], and which differ in their suitability for species dispersal [13], [20].

The persistence of populations of a species is affected by dispersal, because many habitat patches do not contain all the essential resources or are too small to support viable populations [12], [16], [25]. The dispersal ability of a particular species depends on its mobility, but also on habitat quality, landscape structure and landscape connectivity [26–29]. Low connectivity limits dispersal and may lead to a significant loss of genetic variation and even to local extinctions [26], [30], [31]. In classic metapopulation ecology, landscape connectivity is regarded as a property of the spatial structure of discrete habitat patches, while in landscape ecology, connectivity is seen as a property of the entire landscape [19]. Landscape connectivity may be defined as the combined effect of landscape elements that facilitate or disrupt the movement of individuals between patches [28]. For example, a matrix dominated by forest significantly limits the dispersal of grassland butterflies, as indicated by low emigration from natal patches and higher mortality of dispersers, while open environments enhance their dispersal [20].

Successful outcomes of dispersal–such as colonization of vacant patches or, in declining populations, the rescue effect [32]–are also affected by the quality of the habitat patches reached by dispersing individuals. In the butterfly *Parnassius smintheus*, both higher habitat quality and better connectivity of target patches resulted in a higher number of immigration events [33]. In specialist species with strict habitat requirements, the habitat quality of target patches may in fact be the key factor limiting colonization, more important than patch isolation [34], [35]. For insects such as butterflies, the main element of habitat quality is the availability of forage and other required resources [36]. The feeding resource depends on the abundance and distribution of host plants for larvae and nectar plants for imagos [12], [37]. Other key factors include vegetation architecture and habitat components such as shrubs [24], [36]. Components such as invasive plants may also affect habitat quality [7].

To identify the habitat requirements of species and to plan conservation measures, comprehensive studies of the configuration and composition of habitats are needed [11], [19], [28], [38], [39]. The relative roles of habitat patch spatial structure, their quality, and the characteristics of patch surroundings in the success of patch colonization are unclear, and worth an attempt to quantify. We used the dryad butterfly *Minois dryas* (Scopoli, 1763) as a model organism to study the factors driving the colonization of previously unoccupied habitat patches from a species refugium. The focal species inhabits two contrasting habitat types–xerothermic grassland and wet meadow (see [24] for more details), both of which are key habitat types for biodiversity conservation in Europe [40], [41]. To develop effective management strategies on both patch and landscape scales, we need to know which characteristics define the most suitable habitat for the dryad.

Based on metapopulation theory, landscape ecology and resource-based habitat concept, we proposed four hypotheses to explain the patterns of dryad occupancy and abundance across local habitat patches. The occupancy pattern indicates the probability of patch colonization, while the abundance pattern indicates the probability of long-term persistence of newly established local populations. We predicted higher probability of occupancy and higher relative abundance of the focal species for habitat patches that (1) are of good habitat quality (reflected in vegetation structure or resource abundance), (2) have advantageous spatial structure (large area, high connectivity, low level of within-patch fragmentation), and (3) are surrounded by a more hospitable matrix, facilitating dispersal (e.g. lower share of forest, lower road density, higher density of watercourses offering potential corridors), although a higher share of forest on patch edges may positively affect butterfly abundance. Finally, (4) we expected a strong negative effect of distance from the recent species refugium on patch occupancy.

## Methods

### Study area and selection of habitat patches

The study was conducted from July to September 2013 within a large meadow complex in the Vistula River valley, ca. 8 km south-west of the Kraków city centre. The area, covering ca. 35 km^2^, is part of the Bielańsko-Tyniecki Landscape Park and the Dębnicko-Tyniecki Obszar Łąkowy Natura 2000 site (PLH 120065). Field surveys were conducted with a proper permission from the Polish Generalny Dyrektor Ochrony Środowiska No. DOPozgiz.6401.01.38.2011.JRO.2. 18th February 2011. Under Polish law any permission from landowners is not required if the land is not fenced. In the meadow complex a habitat mosaic is formed by xerothermic grassland patches on small calcareous hills and wet meadow patches occupying the flat valley bottom (S1 File). Other habitats or features in the area include degraded meadows (mostly overgrown with reeds, invasive goldenrod or shrubs), fallows, arable land, forests, watercourses, a highway, secondary roads and settlements (S1 File). The western fragment of the study area includes the Skołczanka Nature Reserve, which used to be the only site where the dryad survived in Poland [42], [43]. In recent decades the species has spread from that site to surrounding areas. It inhabits both xerothermic grassland and wet meadow, and its local populations form a classical metapopulation system.

We distinguished 53 habitat patches suitable for the species; all of them were mapped from late August to early September with Garmin 12XL GPS units. Meadows with predominant cover of sedges, bushes, *Geranium pratense* or alien goldenrod were not taken into account. The patches of xerothermic grassland (n = 27) were covered with vegetation of the Festuco-Brometea class or vegetation of thermophilous sandy grassland mainly resembling Festuco-Thymetum serpylli. The patches of wet meadow (n = 26) included meadows with vegetation of the order Molinietalia, mainly Molinion vegetation of different quality, as well as relatively humid lowland hay meadows with *Arrehanterum elatius*, *Phleum pratense* and *Alopecurus pratensis*. The majority of the habitat patches are unmanaged; 15 patches were mown in the year of our study. The mowing regime in the region is consistent; the same patches are mown each year, typically once a year in late June.

### Butterfly survey

The presence and number of dryads were recorded using the standard Pollard walk method [44] on transects 5 m wide in all habitat patches. The length of the transects depended on patch area and was fixed as follows: 50 m for very small patches (< 0.25 ha), 100 m for medium-size patches (0.25–3 ha), 200 m for large patches (3–10 ha), and 500 m for very large patches (> 10 ha). The transects were established as broken lines with ≤ 90° turning angles. Three counts of butterflies were made in each patch between 28 July and 24 August, at approximately two-week intervals, between 11:00 and 17:00 hours on sunny, windless days. An observer moved along the transects at a steady pace of ca. 10 m per 1 min. To make the results comparable for patches (and thus transects) of different sizes, the butterfly abundance indices are expressed as number of butterflies recorded per transect area (length × 5 m width).

### Vegetation study

The general quality of the habitat patches and their vegetation composition were noted at points along the transects, established at randomly generated lengths along the transects (3–10 points per transect depending on transect length). The vegetation within a 1.5-m radius was described for each point. Five measurements of vegetation height, one at the central point and the other four 1.5 m apart to the north, south, east and west, were taken with a measuring tape to 1 cm accuracy. Distance to the nearest shrub was measured to 10 cm accuracy. All nectar plant species within a 1.5-m radius were counted and their ground cover was recorded. Cover was scored on the following scale: 1 = < 10%; 2 = 10–20%, …, 9 = 80–90% and 10 = 90–100%. All together, the vegetation was surveyed at 100 random points for xerothermic grassland patches and 110 points for wet meadow patches, once at each point. The vegetation study was done from mid-July to early August. For a few xerothermic habitat patches it was not possible to determine plant species composition because these patches were completely mown throughout this period.

### Statistical analysis

From field investigations and analysis of GIS maps in Idrisi 2.0 software [45], for each patch we evaluated numerous habitat parameters of potential importance for the dryad (Tables 1 and 2). We differentiated three groups of factors (Table 1). The first group contains variables describing patch habitat quality, such as vegetation height, goldenrod cover, nectar plant cover, shrub density, occurrence of mowing, and habitat type. The second group contains variables describing the spatial characteristics of habitat patches, such as area, fragmentation, connectivity, and distance from species refugium. For variables based on distance (connectivity, distance from refugium), distance was measured from patch centroids. The third group contains variables reflecting landscape composition in the vicinity of a patch. These parameters, including road and watercourse density as well as the percentage of forest in patch surroundings, were calculated for 100-m buffer zones around the patches. Wider buffer zones could not be demarcated because of the short distance between patches; wider buffers would greatly overlap in many cases, making the parameter values derived for them dependent throughout. The dryad is regarded as a sedentary species; 100-m buffer width is probably close to the upper dispersal limit for individuals of this species [46]. We also assessed the percentage of forest in 6-m wide buffers around the patches, as a measure of the proportion of forest at patch edges, but this variable was not included in the analysis due to its strong correlation with the percentage of forest in the 100-m buffers (r = 0.841, P < 0.001). We also excluded several other parameters originally derived from GIS maps, which turned out to be highly correlated with factors already included which performed better as predictors; for example, we excluded the share of built-up areas in the 100-m buffers (correlated with road density) or alternative measures of patch isolation such as distance to nearest other patch or distance to nearest occupied patch (both correlated with connectivity).

**Table 1:** Parameters of habitat patches and their surroundings used as predictors in analyses of the occurrence and abundance patterns of the dryad butterfly. Parameters were classified as reflecting habitat quality (Q), habitat patch spatial structure (S) or characteristics of patch surroundings in 100-m buffers (B).

Variable	Description	Parameter type	Data source
Habitat type	xerothermic grassland or wet meadow (dichotomous variable); expected higher probability of the occurrence and the higher butterfly abundance in xerothermic grasslands	**Q**	Field work
Vegetation height [cm]	a measure of successional stage; expected possible positive effect on the species occurrence and abundance due to higher cover of grasses–potential larval host plants	**Q**	Field work
Mowing	presence or absence of moving in particular habitat fragment; expected negative effect on the species abundance due to application of mowing in inappropriate time and positive effect on the species occurrence (in the long-term perspective)	**Q**	Field work
Shrub density [m^-1^]	a measure of successional stage, approximated as the inverse distance to the nearest shrub; expected positive effect on the species occurrence	**Q**	Field work
Goldenrod cover [1–10]	a measure of invasive plant abundance as goldenrods are the most common alien invasive plants in the region; expected negative effect on the species occurrence and abundance	**Q**	Field work
Nectar plant cover [1–10]	a measure of nectar availability; expected positive effect on the species occurrence and abundance	**Q**	Field work
Patch size [ha]	total area of suitable habitat fragment; expected positive effect on the species occurrence and abundance	**S**	GIS maps
Mean distance from patch interior to edge [m]	mean distance of points within patch from its edge standardised for patch size through dividing by the square root of patch area; adopted as an inverse measure of internal fragmentation of habitat patch; expected positive effect on the species occurrence and abundance	**S**	GIS maps
Patch connectivity	Hanski's connectivity index I_3_ defined as *Σexp(*–*d*_*ij*_ *)* where *d*_*ij*_ is the distance [km] of patch *i* to other patches (*i* ≠ *j*) [84]; adopted as an inverse measure of patch isolation; expected positive effect on the species occurrence and abundance	**S**	GIS maps
Distance from the Skołczanka reserve [m]	a measure of patch isolation from the historical refugium of the species; expected negative effect on the species occurrence and no effect on the species abundance	**S**	GIS maps
% of forest in 100-m buffer	a measure of potential barriers for dispersal; expected negative effect on the species occurrence but positive on the species abundance	**B**	GIS maps
Road density in 100-m buffer [m*ha^-1^]	a measure of potential anthropopressure and barriers for dispersal; expected negative effect on the species occurrence and abundance	**B**	GIS maps
Watercourse density in 100-m buffer [m*ha^-1^]	a measure of availability of potential corridors supporting dispersal and a measure of habitat moisture; expected positive effect on the species occurrence and abundance	**B**	GIS maps

**Table 2:** Correlation coefficients for investigated variables. Significant correlations (*P* < 0.05) are bolded.

Variable	1	2	3	4	5	6	7	8	9	10	11
1. Patch size		-0.206	-0.079	0.143	**-0.291**	0.059	-0.091	0.230	-0.237	0.098	0.091
		*P* = 0.139	*P* = 0.575	*P* = 0.308	***P* = 0.035**	*P* = 0.676	*P* = 0.517	*P* = 0.098	*P* = 0.088	*P* = 0.483	*P* = 0.515
2. % of forest			**-0.280**	**-0.443**	0.153	0.151	-0.111	**-0.394**	**0.365**	**-0.302**	-0.231
			***P* = 0.042**	***P* = 0.001**	*P* = 0.276	*P* = 0.281	*P* = 0.430	***P* = 0.004**	***P* = 0.007**	***P* = 0.028**	*P* = 0.096
3. Road density				-0.138	0.007	-0.265	-0.122	0.130	0.042	0.108	0.135
				*P* = 0.326	*P* = 0.959	*P* = 0.055	*P* = 0.383	*P* = 0.353	*P* = 0.767	*P* = 0.441	*P* = 0.334
4. Watercourse density					-0.025	0.072	-0.063	**0.518**	**-0.286**	**0.397**	-0.197
					*P* = 0.862	*P* = 0.609	*P* = 0.653	***P* < 0.001**	***P* = 0.038**	***P* = 0.003**	*P* = 0.157
5. Mean distance from patch interior to edge						0.176	-0.069	0.121	0.215	0.103	**-0.390**
						*P* = 0.207	*P* = 0.624	*P* = 0.389	*P* = 0.122	*P* = 0.465	***P* = 0.004**
6. Patch connectivity							-0.038	0.107	0.248	0.222	**-0.294**
							*P* = 0.785	*P* = 0.444	*P* = 0.074	*P* = 0.110	***P* = 0.033**
7. Distance from the Skołczanka								**-0.294**	0.040	**-0.329**	**0.277**
								***P* = 0.033**	*P* = 0.775	***P* = 0.016**	***P* = 0.045**
8. Vegetation height									-0.022	**0.618**	-0.129
									*P* = 0.875	***P* < 0.001**	*P* = 0.357
9. Shrub density										0.114	-0.136
										*P* = 0.416	*P* = 0.330
10. Goldenrod cover											-0.257
											*P* = 0.063
11. Nectar plant cover											
											

In analyzing the availability of selected plant types we first assessed their total cover for each random point, and then calculated mean values for the random points in each patch. In this study, by nectar plants we mean all species of groups clearly preferred by the dryad, as found in our earlier research on butterfly feeding preferences [24] as well as single species from other groups at which nectaring was observed (without invasive goldenrod and Apiaceae plants).

To describe habitat patch diversity we performed principal component analysis (PCA), a linear unconstrained ordination method [47], using selected environmental variables. Because PCA is sensitive to numerical values and may lead to incorrect results [48], all the variables were standardized by dividing the values of environmental variables for each patch by the means for all the habitat patches. We used PERMANOVA to test whether grouping factors (habitat type, dryad presence-absence, and their interaction term) have significant effects on patch characteristics [49]. The analysis used Euclidean distance, which is more appropriate for standardized data [48].

In the main analysis we tested the factors affecting the occurrence and abundance of the dryad in habitat patches. Based on the species detection records from three consecutive counts along the transects, we calculated detectability using the occupancy model of MacKenzie et al. [50] in the Mark program [51]. The model gives estimates of species detection probability (*p*) and site occupancy rate (the fraction of sites occupied by the species) (*ψ*), using a maximum likelihood approach. Its rationale follows that of mark-release-recapture (MRR) surveys, but individuals are replaced by sites surveyed. At each site, species presence is checked several times, resulting in a site-specific species-detection history, with 1 indicating detection and 0 indicating non-detection. The crucial assumption is that the system is closed; that is, there are no changes in species presence-absence patterns during the survey. Our protocol ensured this by having all three transect counts done within the flight period of the dryad. Although an optimal sampling design should consist of at least 20 sites and at least 5 sampling occasions, for butterflies 2 or 3 sampling occasions should be enough in view of their ease of detection [52], [53].

The outcome of the model revealed very high detectability, reaching 90% for a single transect count. This means that the estimated proportion of occupied sites only marginally exceeded the proportion of patches in which the species was detected during the surveys: the chance that one more patch was actually occupied by the dryad was ca. 2.5%. Consequently, we treated the recorded presence-absence of the dryad as its true presence-absence. Its abundance is expressed as the total number of individuals from three counts per 1 ha transect. To achieve normality of that parameter we applied cubic-root transformation in all the analyses. We found no spatial autocorrelation of local abundance of the dryad among the investigated patches.

In order to test the effects of habitat parameters on dryad occupancy and abundance in the occupied patches, we used model selection and model averaging procedures based on information theory [54]. We applied the Akaike information criterion corrected for small sample size (AIC_c_) to find the set of the most supported models among all possible models. We ranked all the models built according to their ΔAIC_c_ values, where ΔAIC_c_ is the difference between a given model and the one with the lowest AIC_c_, and we defined as supported all the models with ΔAIC_c_ < 7 [54] (Tables A and B in S2 File). Then we averaged the model results across all the supported models, using their Akaike weights, which reflect the probability that a given model is the best one. We assessed the relative importance of each explanatory variable by calculating the cumulative Akaike weights of models containing a particular variable [54]. In models built for dryad occupancy pattern, we adopted logistic regressions and binomial distributions of the dependent variable, while in those for species abundance pattern we used the general linear model (GLM) approach and Gaussian distributions of the dependent variable.

From the set of independent variables we excluded those highly correlated with others that performed better as predictors (see above). The parameters tested in the models were only moderately correlated with each other, having Pearson’s r below 0.5 (Table 2) and thus within the range in which variables in multivariate analyses may be regarded as independent [55]. Vegetation height was the exception but we decided to keep this parameter because it performed relatively well as a predictor, including in the models that contained the two other factors with which it correlated. In the case of watercourse density, vegetation height, and cover of nectar plants, for which different effects might be expected in xerothermic grassland and wet meadow, we also tested their interactions with habitat type. However, we applied stepwise backward elimination of the nonsignificant interactions, and the final models retained only the statistically significant interactions.

As distance from the refugium turned out to be the predominant factor limiting species occurrence, with the farthest occupied patch lying ca. 2300 m away (see results), we also conducted an additional analysis restricted to patches located within this threshold distance from the refugium. However, due to the small sample size (only 5 unoccupied and 18 occupied patches) it was not possible to fit multifactorial models, so we performed a model selection routine for models with only a single explanatory variable.

Correlation analysis was performed in Statistica 10.0, PCA in Canoco for Windows 4.5 [47], and PERMANOVA analysis in PAST 3.01 [56]. Model selection and averaging procedures were run in R 3.0.2 for Windows using the Stats and MuMIn packages [57].

## Results

Dryads were observed in only 9 patches of xerothermic grassland and 9 patches of wet meadow (S1 File). The other 35 meadows were not occupied by the species. We recorded 149 adult butterflies in xerothermic grassland and 157 adult butterflies in wet meadow. The dryad’s mean relative abundance per 1 ha of transect in occupied patches was estimated at 435.6 (± 61.17 SE) in xerothermic grassland patches and 418.2 (± 178.90 SE) in wet meadow patches.

### Characteristics of habitat patches

For the investigated environmental variables, four PCA axes explained 87.8% of the variance of habitat patch diversity. The first ordination axis explained 35.3%, the second axis 25.6%, the third axis 14.8% and the fourth axis 12.1% of overall variance.

**Fig 1:**
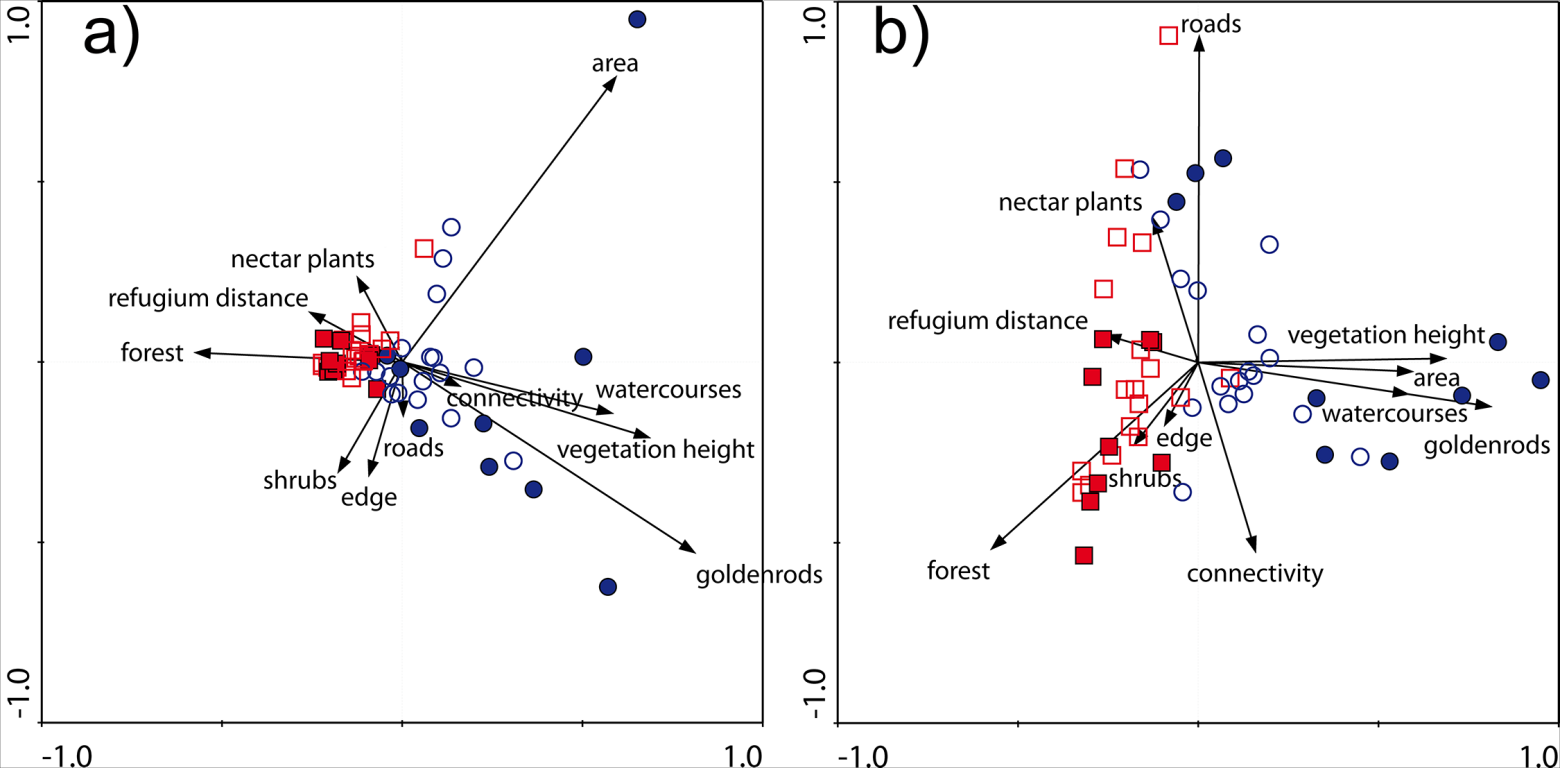
PCA ordination of the investigated patch characteristics. Plate a–first and second ordination axes; Plate b–first and third ordination axes; squares–xerothermic grasslands; circles–wet meadows; filled symbols–occupied habitat patches; open symbols–vacant habitat patches. Key to variables: area–patch size, edge–mean distance from patch interior to edge, forest–% of forest, goldenrods–goldenrod cover, connectivity–patch connectivity, nectar plants–nectar plant cover, refugium distance–distance from the Skołczanka reserve, roads–road density, shrubs–shrub density, vegetation height–vegetation height, watercourses–watercourse density

Xerothermic grassland differed significantly from wet meadow (*F* = 14.087, *P* << 0.001). The xerothermic grassland patches were characterized by higher abundance of nectar resources and higher shares of forest in their surroundings; they were also slightly more fragmented, and a larger proportion of those patches was mown (Fig 1). The wet meadow patches were larger, had higher density of watercourses in their surroundings, taller vegetation, and more cover of invasive goldenrod. The wet meadow patches were also less isolated, as reflected in a higher Hanski’s connectivity index (Fig 1 and S1 File).

Occupied patches differed significantly from unoccupied patches (*F* = 4.592, *P* < 0.001); the interaction of habitat type and dryad presence was also statistically significant (*F* = 4.807, *P* < 0.001). The occupied xerothermic grassland patches were smaller, less isolated, had larger shares of forest and lower density of roads in their surroundings, and had less nectar resources than the unoccupied xerothermic grassland patches (Fig 1). Occupied wet meadow patches were less isolated, larger, and had more goldenrod cover, taller vegetation, slightly higher density of watercourses and higher density of shrubs than the unoccupied wet meadows (Fig 1). The maximum goldenrod cover recorded from occupied patches was 33% (Table C in S2 File). The characteristics of habitat patches and the PCA scores for environmental variables are presented in detail in Tables C and D in S2 File.

### Factors affecting the occurrence and density of the dryad in habitat patches

The model selection procedure indicated that the occurrence of the dryad in our study area is strongly influenced by distance from the Skołczanka Reserve, which was a predictor in all supported models (Table A in S2 File). The probability of patch occupancy clearly decreased with the distance from this refugium (Table 3). The farthest occupied patch was 1640 m distant from the Skołczanka Reserve (S1 File); more distant patches were vacant. Shrub density, mowing and goldenrod cover may also play roles in the dryad’s occurrence. They all appeared in the models with the lowest AIC_c_ and are important in the set of supported models, as indicated by their cumulative weight (Table A in S2 File and Table 3; see Methods for details of the procedure). On the other hand, none of the above factors reached statistical significance in the model-weighted outcomes of the analysis, although the effect of shrub density was close to significant, and the analysis that included patches located up to 2300 m from the species refugium showed a strong positive effect of shrub density (estimate = 24.120 ± 11.240 SE, *P* = 0.032, cumulative weight = 0.91) and a slight positive effect of patch connectivity (estimate = 0.541 ± 0.253 SE, *P* = 0.033, cumulative weight = 0.06) on dryad occurrence.

**Table 3:** Outcome of analysis of factors affecting dryad occurrence in habitat patches. Table shows weighted average results for all models calculated using model Akaike weights. Significant results are bolded.

	Cumulative weight	Estimate	Adjusted SE	*z*	*P*
(Intercept)		5.348	3.377	1.875	0.095
Distance from the Skołczanka reserve	**1.00**	**-0.004**	**0.002**	**2.491**	**0.013**
Shrub density	0.42	13.237	7.619	1.737	0.082
Mowing (yes)	0.18	1.960	2.789	0.703	0.482
Goldenrod cover	0.14	0.472	1.704	0.277	0.782
Mean distance from patch interior to edge	0.12	-49.470	44.327	1.116	0.264
Road density	0.08	0.009	0.013	0.676	0.499
Patch size	0.07	0.345	1.043	0.331	0.741
Patch connectivity	0.05	-0.180	0.496	0.363	0.716
Nectar plant cover	0.05	0.112	0.299	0.376	0.707
Habitat type (wet)	0.05	-0.502	1.571	0.319	0.750
Vegetation height	0.05	0.011	0.029	0.365	0.715
Watercourse density	0.04	-0.003	0.016	0.178	0.859
% of forest	0.04	-0.510	2.905	0.176	0.861

The weighted-average results of the models explaining the patterns of dryad abundance in occupied patches showed significant negative effects of patch size, patch compactness, and invasive goldenrod cover, as well as a significant positive effect of the density of watercourses in the 100-m patch buffers (Table B in S2 File and Table 4). The effect of nectar plant cover was modified by its significant interaction with habitat type. In xerothermic grassland patches, dryad abundance was positively related to the availability of nectar plants, while in wet grassland patches it was negatively affected by the same factor (Table B in S2 File and Table 4).

**Table 4:** Outcome of analysis of factors affecting dryad abundance in habitat patches. Table shows weighted average results for all models calculated using model Akaike weights. Significant results are bolded.

	Cumulative weight	Estimate	Adjusted SE	*z*	*P*
(Intercept)		11.880	4.097	2.901	0.004
Goldenrod cover	**0.98**	**-1.528**	**0.521**	**2.934**	**0.003**
Watercourse density	**0.90**	**0.025**	**0.010**	**2.386**	**0.017**
Nectar plant cover	0.84	-0.356	0.193	1.841	0.066
Patch size	**0.58**	**-0.284**	**0.120**	**2.378**	**0.017**
Mean distance from patch interior to edge	**0.54**	**-37.280**	**14.190**	**2.627**	**0.009**
Habitat type (wet)	0.34	2.431	4.859	0.500	0.617
Habitat type (wet):nectar plant cover	**0.19**	**-0.742**	**0.286**	**2.591**	**0.010**
Mowing (yes)	0.12	-1.268	0.856	1.481	0.139
Vegetation height	0.05	0.028	0.026	1.074	0.283
Patch connectivity	0.03	-0.147	0.186	0.791	0.429
% of forest	0.02	0.570	1.161	0.491	0.623
Road density	0.01	-0.002	0.005	0.328	0.743
Shrub density	0.01	-0.534	1.966	0.272	0.786
Distance from the Skołczanka reserve	0.01	0.000	0.001	0.029	0.977

## Discussion

Here we demonstrated that colonization of the studied habitat patches by the specialist dryad butterfly is strongly limited by the distance from its recent species refugium, the Skołczanka Reserve. The second factor apparently facilitating colonization is shrub density, which positively affects dryad occurrence; matrix composition appears to be of less importance. This implies that grasslands experiencing early successional stages of shrub intrusion are most suitable for the dryad. Characteristics reflecting patch quality are likely to affect the persistence of already established populations by shaping their local abundance. Generally, our results are in agreement with earlier findings on wetland butterflies from a similar study system [35], but the relative importance of the factors was different. We found that habitat quality is less important than distance from the refugium in limiting colonization by the dryad butterfly. Our results also suggest that the dryad has rather low dispersal ability.

The occurrence of the dryad was affected most directly by a single factor, distance from the Skołczanka refugium. Landscape structure, particularly the matrix surrounding the patches, did not affect its distribution. Density of watercourses did not influence its occurrence either, contrary to our expectations, as waterways might be expected to act as corridors for butterfly dispersal. However, watercourse density in patch surroundings turned out to have a beneficial effect on habitat quality. It positively affected dryad abundance, suggesting that the dryad needs humid habitat fragments, especially in xerothermic grassland. Sites with a more humid microclimate support the growth of grasses, which are foodplants of the dryad; such habitat patches can be expected to support more viable local populations.

Other factors reflecting habitat quality may also shape the dryad’s occurrence in different areas of its Euro-Siberian range [42], especially in metapopulations that are in equilibrium rather than being shaped by recent colonization processes. A study of the dryad in Japan revealed the importance of forest/grassland edges for facilitation of immigration [58]. This implies that, against our expectations, forests surrounding habitat patches do not necessarily function as a barrier to the dryad. In fact, Akeboshi et al. [58] interpret this type of edge as a factor improving patch quality. It has been shown that the occurrence of many butterfly species is driven mainly by habitat quality factors, including management regimes, plant species composition and larval host plants [13], [59], [60]. Habitat spatial structure parameters such as patch isolation or patch size, and barriers such as forests, are mostly unimportant in this respect [13], [37], [59], [61], although for some butterflies the spatial structure of habitats and the characteristics of the matrix have been found to matter [20], [61], [62].

Our results suggest that the dryad’s colonization of the study area is very recent and that further spread of the species to new and previously occupied sites is highly likely. We discovered that the dryad occupied non-optimal habitat patches with a relatively high share of invasive plants and degraded plant communities. We found a strong negative impact of invasive alien goldenrod on dryad abundance but not on its occurrence. It appears that even relatively high cover of invasive goldenrod does not prevent colonization but may hamper the long-term persistence of established populations by limiting their size. Despite the negative impact of goldenrod on butterfly abundance, patches invaded by these alien plants were still inhabited by the dryad. We suggest that some relaxation of its habitat requirements appears to have promoted the dryad’s recent spread. In another case, the habitat requirements of *Lycaena dispar* butterfly have loosened; the species is using drier habitats and new species of foodplants [63]. In extreme cases, high cover of goldenrod in small patches may seriously reduce the availability of larval host grasses of the dryad and threaten its persistence, due to an insufficiency of vital resources and a consequent high risk of a stochastic extinction cf. [12], [16]. We noted two such meadows fairly close to the Skołczanka Reserve; they were in fact the only vacant patches in the vicinity of this refugium (authors’ unpubl. data). In general, alien plants are a great threat to biodiversity; the negative impact of invasive goldenrod has been reported for many species [7], [64], [65]. In an earlier study of the dryad we found such an effect on within-patch habitat use by adult butterflies [24], and the present research on its patterns of local abundance confirms this effect on a much larger scale.

In both xerothermic grassland and wet meadow, the habitat quality of dryad habitat patches was shaped mostly by the same environmental gradients related to plant species composition. In our detailed analysis, however, we found one index of habitat quality that differed in its effect on dryad abundance between the two habitat types–availability of nectar plants. Unexpectedly, greater nectar plant cover translated to lower dryad abundance in wet meadows, while in xerothermic grassland patches the reverse was true. This implies that dryad can persist in low quality patches only if they offer basic resources in the form of common grasses serving larval host plants, and minimal availability of nectar plants. This result is in line with our previous findings [24], and suggests that xerothermic grassland is a better habitat for adult butterflies due to the greater availability of nectar plants, while wet meadows, with more abundant grasses used as foodplants, are more suitable for caterpillars. Many nectar plants offered by wet meadow are in fact plants avoided by the dryad (e.g. *Fabaceae*) [24]. The unexpected effect of nectar plants may be related to natural succession; lower availability of nectar plants is often related to taller vegetation, which provides shelter for adult butterflies.

Apart from habitat composition, factors describing habitat patch spatial structure also play some role for the dryad. Its abundance increased with the increase of within-patch fragmentation, as indicated by the negative effect of mean distance of patch interior from patch edge, as well as with decreasing patch size. That the relative density of the dryad was greater in smaller and more fragmented patches may seem surprising at first glance, but it is rather typical for butterflies [66]. Such a pattern apparently reflects the higher average quality of small occupied patches; small low-quality patches are unlikely to support local populations, as predicted by metapopulation theory [16]. In addition, higher species density in small fragmented patches may reflect some positive influence of patch edges cf. [41]. We suggest that the dryad benefits from edges due to the presence of bushes and tall vegetation there, which in our earlier work was found to be used as resting sites [24]. We have observed dryads resting on patch boundaries with forest, scrub and reedbeds on hot days (authors’ unpublished data). The higher dryad abundance in patches with higher within-patch fragmentation supports the behaviour-at-boundaries hypothesis, according to which habitat specialists tend to return more frequently to the interior of patches at habitat boundaries than a more mobile habitat generalist does [67].

### Conservation perspectives

The dryad is a relatively large, oligophagous butterfly with one-year development, strict habitat requirements, and poor mobility [46], [68]. Our findings confirm that this species is a habitat quality-based butterfly; the long-term persistence of colonizing populations strongly depends on the quality of target patches. The matrix composition is of less importance to it. Management measures aimed at supporting dryad colonization of vacant patches must be adapted to the habitat type. Optimally, vegetation height for the dryad should be low in xerothermic grassland but higher in wet meadow. These guidelines translate to different recommended mowing regimes for the two habitat types. As mowing turned out to have a slight negative impact on dryad abundance in the short term, a rotational mowing regime will help to minimize this effect [69]. In xerothermic grassland, rotational mowing should be done in early spring. In wet meadow a single fragment of a habitat patch or a whole patch area should be mown every 2–3 years in autumn, mid-September at the earliest [70]. In large patches, fragments that clearly differ from the typical plant species composition of Molinion (e.g. fragments dominated by *Filipendula ulmaria*) can be mown more frequently. Invasive goldenrod should be removed in early summer before flowering, as we previously suggested [24]. Goldenrod cover at sites inhabited by the dryad should not exceed 33% of the vegetation structure; this should enable the butterfly populations to persist despite the negative impact of goldenrod on dryad abundance.

At landscape scale, the biggest threat to dryad populations inhabiting wet meadows is desiccation of these habitats through ill-conceived drainage work. Land managers should prevent such work around dryad sites in order to maintain high watercourse density, and should monitor the soil moisture of these sites.

To safeguard metapopulations, the largest habitat patches should be preserved, as a means of ensuring the continuity of local populations [16]. It is likely that a source-sink system operates in our study area; the smallest habitat patches may be too small to sustain viable local populations. Due to differences in the conditions and distribution of resources, as well as differences in their influence on the dryad (e.g. opposite effects of nectar plant cover on dryad abundance) in contrasting habitat types, this source-sink dynamic, if it occurs, probably operates in only one habitat type cf. [71].

On the other hand, as the smaller and more fragmented patches had higher dryad density, their role in metapopulation functioning should not be neglected. Specialist species are thought to require habitats of good quality, with specific vegetation well developed and not overgrown with shrubs e.g. [72], [73]. Recently, Yan Chong et al. [74] demonstrated that cultivated and artificial greenery in an urban landscape supports lower butterfly community diversity than natural and semi-natural vegetation does.

Our findings make it clear that even partially degraded patches of semi-natural grassland of lower quality (invaded by goldenrod, having degraded plant species communities, having higher within-patch fragmentation) are conducive to the persistence of species with specific habitat requirements. Our work also underlines the importance of small open and shrubby patches in supporting dryad dispersal and presence, facilitating its long-term conservation. The same principles may apply in the conservation of other butterflies with similar requirements, such as *Hamearis lucina*, *Eerebia aethiops* or *Leptidea sinapis*, which depend on small shrubby patches of grassland [75–77]. We conclude that such sites are essential for maintaining biodiversity, particularly in urban areas cf. [78]. Small patches with convoluted edges may function as stepping-stones, facilitating species dispersal and turnover of individuals [79–81], especially under conditions of positive density-dependent dispersal [82], [83].

## Supporting Information

S1 File Location and spatial structure of the study area in the Kraków region, southern Poland. Yellow–xerothermic grassland patches; blue–wet meadow patches; red lines–boundaries of patches occupied by the dryad; bolded red lines–boundaries of the species refugium, the Skołczanka Nature Reserve. Data are presented in the EPSG coordinate reference system (32634 –WGS 84 / UTM zone 34N).
(KML)

S2 File Characteristics of habitat patches and their impact on the dryad. Table A. Supported models describing the occurrence of the dryad in habitat patches. For each model we list the codes of the predictors included (k), log-likelihood (logLik), the Akaike information criterion value (AIC_c_) together its difference from the AIC_c_ of the best model (Delta), and Akaike weight (Weight). Predictor codes: 1 –Patch size; 2 –% of forest; 3 –Road density; 4 –Watercourse density; 5 –Mean distance from patch interior to edge; 6 –Patch connectivity; 7 –Distance from the Skołczanka; 8 –Vegetation height; 9 –Shrub density; 10 –Goldenrod cover; 11 –Nectar plants cover; 12 –Habitat type; 13 –Mowing; Table B. Supported models describing the abundance of the dryad in habitat patches. For each model we list the codes of the predictors included (k), log-likelihood (logLik), the Akaike information criterion value (AIC_c_) together its difference from the AIC_c_ of the best model (Delta), and Akaike weight (Weight). Predictor codes: 1 –Patch size; 2 –% of forest; 3 –Road density; 4 –Watercourse density; 5 –Mean distance from patch interior to edge; 6 –Patch connectivity; 7 –Distance from the Skołczanka; 8 –Vegetation height; 9 –Shrub density; 10 –Goldenrod cover; 11 –Nectar plants cover; 12 –Habitat type; 13 –Mowing; 14 –Habitat type:nectar plant cover; Table C. Basic characteristics of habitat patches of xerothermic grassland and wet meadow occupied or not occupied by the dryad in the study landscape; Table D. Results of PCA analysis giving environmental variable scores for each ordination axis. Significant contributions are bolded.
(DOCX)
