# Supplementary material for: How Do Landscape Structure, Management and Habitat Quality Drive the Colonization of Habitat Patches by the Dryad Butterfly (Lepidoptera: Satyrinae) in Fragmented Grassland?
Source: PLoS One. 2015 Sep 16;10(9):e0138557. doi: 10.1371/journal.pone.0138557 (PMC4573758; doi:10.1371/journal.pone.0138557)
Supplement: S2 File — Table A. Supported models describing the occurrence of the dryad in habitat patches. For each model we list the codes of the predictors included (k), log-likelihood (logLik), the Akaike information criterion value (AICc) together its difference from the AICc of the best model (Delta), and Akaike weight (Weight). Predictor codes: 1 –Patch size; 2 –% of forest; 3 –Road density; 4 –Watercourse density; 5 –Mean distance from patch interior to edge; 6 –Patch connectivity; 7 –Distance from the Skołczanka; 8 –Vegetation height; 9 –Shrub density; 10 –Goldenrod cover; 11 –Nectar plants cover; 12 –Habitat type; 13 –Mowing; Table B. Supported models describing the abundance of the dryad in habitat patches. For each model we list the codes of the predictors included (k), log-likelihood (logLik), the Akaike information criterion value (AICc) together its difference from the AICc of the best model (Delta), and Akaike weight (Weight). Predictor codes: 1 –Patch size; 2 –% of forest; 3 –Road density; 4 –Watercourse density; 5 –Mean distance from patch interior to edge; 6 –Patch connectivity; 7 –Distance from the Skołczanka; 8 –Vegetation height; 9 –Shrub density; 10 –Goldenrod cover; 11 –Nectar plants cover; 12 –Habitat type; 13 –Mowing; 14 –Habitat type:nectar plant cover; Table C. Basic characteristics of habitat patches of xerothermic grassland and wet meadow occupied or not occupied by the dryad in the study landscape; Table D. Results of PCA analysis giving environmental variable scores for each ordination axis. Significant contributions are bolded. (DOCX) [file pone.0138557.s002.docx]

**S2 File.** **Characteristics of habitat patches and their impact on the dryad.**

Table A. Supported models describing the occurrence of the dryad in habitat patches. For each model we list the codes of the predictors included (k), log*-*likelihood (logLik), the Akaike information criterion value (AIC_c_) together its difference from the AIC_c_ of the best model (Delta), and Akaike weight (Weight). Predictor codes: 1 – Patch size; 2 – % of forest; 3 – Road density; 4 – Watercourse density; 5 – Mean distance from patch interior to edge; 6 – Patch connectivity; 7 – Distance from the Skołczanka; 8 – Vegetation height; 9 – Shrub density; 10 – Goldenrod cover; 11 – Nectar plants cover; 12 – Habitat type; 13 – Mowing

| k | df | logLik | AIC_c_ | Delta | Weight |
| --- | --- | --- | --- | --- | --- |
| 7;9 | 3 | -4.86 | 16.21 | 0.00 | 0.22 |
| 7;9;13 | 4 | -4.52 | 17.86 | 1.65 | 0.10 |
| 7 | 2 | -7.02 | 18.28 | 2.07 | 0.08 |
| 7;9;10 | 4 | -4.83 | 18.50 | 2.29 | 0.07 |
| 5;7 | 3 | -6.08 | 18.65 | 2.44 | 0.07 |
| 7;13 | 3 | -6.65 | 19.78 | 3.57 | 0.04 |
| 3;7 | 3 | -6.68 | 19.86 | 3.65 | 0.04 |
| 1;7 | 3 | -6.86 | 20.21 | 4.00 | 0.03 |
| 7;9;10;13 | 5 | -4.47 | 20.22 | 4.01 | 0.03 |
| 6;7 | 3 | -6.91 | 20.31 | 4.10 | 0.03 |
| 7;10 | 3 | -6.92 | 20.32 | 4.11 | 0.03 |
| 7;11 | 3 | -6.93 | 20.36 | 4.15 | 0.03 |
| 7;12 | 3 | -6.96 | 20.40 | 4.19 | 0.03 |
| 7;8 | 3 | -6.96 | 20.40 | 4.19 | 0.03 |
| 2;7 | 3 | -7.01 | 20.50 | 4.29 | 0.03 |
| 4;7 | 3 | -7.01 | 20.52 | 4.31 | 0.03 |
| 3;5;7 | 4 | -5.95 | 20.73 | 4.52 | 0.02 |
| 1;5;7 | 4 | -6.03 | 20.90 | 4.69 | 0.02 |
| 7;10;13 | 4 | -6.45 | 21.73 | 5.52 | 0.01 |
| 1;3;7 | 4 | -6.48 | 21.80 | 5.59 | 0.01 |
| 7;11;12 | 4 | -6.85 | 22.53 | 6.32 | 0.01 |
| 6;7;11 | 4 | -6.88 | 22.59 | 6.38 | 0.01 |
| 7;6;12 | 4 | -6.90 | 22.63 | 6.42 | 0.01 |
| 4;7;8 | 4 | -6.91 | 22.66 | 6.45 | 0.01 |
| 2;7;8 | 4 | -6.94 | 22.72 | 6.51 | 0.01 |
| 2;4;7 | 4 | -6.99 | 22.82 | 6.61 | 0.01 |
| 3;5;7 | 5 | -5.90 | 23.08 | 6.87 | 0.01 |

| Table B. Supported models describing the abundance of the dryad in habitat patches. For each model we list the codes of the predictors included (k), log*-*likelihood (logLik), the Akaike information criterion value (AIC_c_) together its difference from the AIC_c_ of the best model (Delta), and Akaike weight (Weight). Predictor codes: 1 – Patch size; 2 – % of forest; 3 – Road density; 4 – Watercourse density; 5 – Mean distance from patch interior to edge; 6 – Patch connectivity; 7 – Distance from the Skołczanka; 8 – Vegetation height; 9 – Shrub density; 10 – Goldenrod cover; 11 – Nectar plants cover; 12 – Habitat type; 13 – Mowing; 14 – Habitat type:nectar plant cover | | | | | | |
| --- | --- | --- | --- | --- | --- | --- |
| k | df | logLik | AIC_c_ | Delta | Weight |  |
| 1;4;5;10;11; | 7 | -20.93 | 67.06 | 0.00 | 0.36 |  |
| 4;10;11;12;14 | 7 | -22.39 | 69.98 | 2.91 | 0.08 |  |
| 10;11;12;14 | 6 | -25.48 | 70.60 | 3.54 | 0.06 |  |
| 4;10;11 | 5 | -27.84 | 70.68 | 3.61 | 0.06 |  |
| 4;10;13 | 5 | -28.26 | 71.52 | 4.45 | 0.04 |  |
| 4;10;12 | 5 | -28.31 | 71.62 | 4.55 | 0.04 |  |
| 4;10;12;13 | 6 | -26.14 | 71.91 | 4.85 | 0.03 |  |
| 1;4;5;10;11;13 | 8 | -20.05 | 72.10 | 5.04 | 0.03 |  |
| 1;4;5;8;10;11 | 8 | -20.17 | 72.34 | 5.27 | 0.03 |  |
| 4;5;10;11 | 6 | -26.37 | 72.37 | 5.31 | 0.03 |  |
| 4;10;8;12 | 6 | -26.47 | 72.58 | 5.52 | 0.02 |  |
| 1;4;10;11 | 6 | -26.50 | 72.64 | 5.58 | 0.02 |  |
| 1;5;10;11;12;14 | 8 | -20.42 | 72.85 | 5.78 | 0.02 |  |
| 4;12 | 4 | -30.92 | 72.91 | 5.85 | 0.02 |  |
| 4;10;11;12 | 6 | -26.66 | 72.95 | 5.89 | 0.02 |  |
| 1;10;11;12;14 | 7 | -23.88 | 72.96 | 5.90 | 0.02 |  |
| 4;6;10;11 | 6 | -26.83 | 73.30 | 6.24 | 0.02 |  |
| 1;4;5;6;10;11 | 8 | -20.66 | 73.33 | 6.26 | 0.02 |  |
| 1;2;4;5;10;11 | 8 | -20.69 | 73.37 | 6.31 | 0.02 |  |
| 1;3;4;5;10;11 | 8 | -20.82 | 73.64 | 6.58 | 0.01 |  |
| 1;4;5;9;10;11 | 8 | -20.86 | 73.71 | 6.65 | 0.01 |  |
| 1;4;5;10;11;12 | 8 | -20.90 | 73.79 | 6.73 | 0.01 |  |
| 1;4;5;7;10;11 | 8 | -20.93 | 73.86 | 6.80 | 0.01 |  |
| 1;4;10;13 | 6 | -27.15 | 73.94 | 6.88 | 0.01 |  |
| 1;4;10;11;12;14 | 8 | -21.00 | 74.01 | 6.95 | 0.01 |  |
| 4;10;11;13 | 6 | -27.21 | 74.05 | 6.98 | 0.01 |  |

Table C. Basic characteristics of habitat patches of xerothermic grasslands and wet meadows occupied or unoccupied by the dryad in the study landscape.

|  |  | |  | |  |  |  |  |  | |  |  |  |
| --- | --- | --- | --- | --- | --- | --- | --- | --- | --- | --- | --- | --- | --- |
| Vacant xerothermic grasslands | | | | |  |  |  | Occupied xerothermic grasslands | | |  |  |  |
|  | | Mean | | Minimum | Maximum | SE |  |  | | Mean | Minimum | Maximum | SE |
| Patch size | | 0.5750 | | 0.024 | 3.452 | 0.19170 |  | Patch size | | 0.3378 | 0.06800 | 0.868 | 0.10250 |
| % of forest | | 0.269 | | 0.00 | 0.62 | 0.0479 |  | % of forest | | 0.502 | 0.00 | 1.00 | 0.0984 |
| Road density | | 45.955 | | 0.00 | 254.93 | 17.4841 |  | Road density | | 29.337 | 0.00 | 90.28 | 11.2752 |
| Watercourse density | | 10.320 | | 0.00 | 97.30 | 5.6405 |  | Watercourse density | | 8.191 | 0.00 | 39.06 | 5.3717 |
| Mean distance from patch interior to edge | | 0.1300 | | 0.100 | 0.194 | 0.00630 |  | Mean distance from patch interior to edge | | 0.1347 | 0.104 | 0.176 | 0.00900 |
| Patch connectivity | | 10.210 | | 3.34 | 13.20 | 0.6874 |  | Patch connectivity | | 9.519 | 6.23 | 12.64 | 0.8219 |
| Distance from the Skołczanka | | 3729.5 | | 408 | 4772 | 235.68 |  | Distance from the Skołczanka | | 680.0 | 0 | 1640 | 171.28 |
| Vegetation height | | 35.13 | | 9.4 | 51.7 | 3.068 |  | Vegetation height | | 47.00 | 37.7 | 57.1 | 2.201 |
| Shrub density | | 0.246 | | 0.0000 | 0.435 | 0.0285 |  | Shrub density | | 0.2972 | 0.11765 | 0.532 | 0.0484 |
| Goldenrod cover | | 0.00 | | 0.0 | 0.0 | 0.000 |  | Goldenrod cover | | 0.04 | 0.0 | 0.3 | 0.037 |
| Nectar plant cover | | 8.91 | | 1.6 | 13.3 | 0.952 |  | Nectar plant cover | | 6.13 | 3.0 | 11.4 | 0.938 |
|  | |  | |  |  |  |  |  | |  |  |  |  |
|  | |  | |  |  |  |  |  | |  |  |  |  |
| Vacant wet meadows | |  | |  |  |  |  | Occupied wet meadows | |  |  |  |  |
|  | | Mean | | Minimum | Maximum | SE |  |  | | Mean | Minimum | Maximum | SE |
| Patch size | | 1.0510 | | 0.066 | 4.154 | 0.29060 |  | Patch size | | 2.0722 | 0.117 | 11.749 | 1.28230 |
| % of forest | | 0.042 | | 0.00 | 0.53 | 0.0314 |  | % of forest | | 0.040 | 0.00 | 0.14 | 0.0202 |
| Road density | | 34.982 | | 0.00 | 140.20 | 11.0735 |  | Road density | | 81.899 | 0.00 | 180.67 | 23.9829 |
| Watercourse density | | 75.253 | | 0.00 | 150.60 | 10.2592 |  | Watercourse density | | 75.634 | 0.00 | 169.64 | 17.1243 |
| Mean distance from patch interior to edge | | 0.1380 | | 0.093 | 0.183 | 0.00640 |  | Mean distance from patch interior to edge | | 0.1339 | 0.088 | 0.171 | 0.00862 |
| Patch connectivity | | 9.127 | | 3.77 | 12.76 | 0.6694 |  | Patch connectivity | | 11.811 | 10.58 | 12.83 | 0.2843 |
| Distance from the Skołczanka | | 3158.7 | | 1524 | 4870 | 222.75 |  | Distance from the Skołczanka | | 611.4 | 312 | 1011 | 83.35 |
| Vegetation height | | 75.39 | | 36.5 | 110.7 | 4.958 |  | Vegetation height | | 85.73 | 39.5 | 122.9 | 9.001 |
| Shrub density | | 0.135 | | 0.00 | 0.80 | 0.049 |  | Shrub density | | 0.227 | 0.05 | 0.79 | 0.07590 |
| Goldenrod cover | | 0.40 | | 0.0 | 1.8 | 0.114 |  | Goldenrod cover | | 1.27 | 0.0 | 3.3 | 0.360 |
| Nectar plant cover | | 7.44 | | 4.3 | 11.0 | 0.486 |  | Nectar plant cover | | 6.65 | 2.7 | 9.4 | 0.916 |

Table D. Results of the PCA analysis representing environmental variable scores for each ordination axis. Significant contributions are presented in bold.

| **Variable** | **Axis 1** | **Axis 2** | **Axis 3** | **Axis 4** |
| --- | --- | --- | --- | --- |
| Patch size  % of forest  Road density  Watercourse density  Mean distance from patch interior to edge  Patch connectivity  Distance from the Skołczanka  Vegetation height  Shrub density  Goldenrod cover  Nectar plant cover | **0.5946** | **0.7853** | **-**0.0237 | 0.1678 |
|  | -**0.5710** | 0.0246 | -**0.5208** | **0.5231** |
|  | 0.0031 | **-**0.1547 | **0.8981** | 0.3600 |
|  | **0.5795** | **-**0.1412 | **-**0.0841 | -**0.6156** |
|  | **-**0.0927 | **-**0.3130 | **-**0.1739 | 0.0155 |
|  | 0.1575 | **-**0.0630 | -**0.5219** | 0.0060 |
|  | **-**0.2544 | 0.1411 | 0.0801 | **-**0.3654 |
|  | **0.6822** | **-**0.2097 | 0.0111 | **-**0.0634 |
|  | **-**0.1769 | **-**0.3045 | **-**0.2320 | **0.4943** |
|  | **0.8075** | **-0.5315** | **-**0.1264 | 0.2036 |
|  | **-**0.1230 | 0.2366 | 0.3993 | **-**0.0390 |
